# An electricity smart meter dataset of Spanish households: insights into consumption patterns

**DOI:** 10.1038/s41597-023-02846-0

**Published:** 2024-01-10

**Authors:** Carlos Quesada, Leire Astigarraga, Chris Merveille, Cruz E. Borges

**Affiliations:** 1 Deusto Institute of Technology, Faculty of Engineering, University of Deusto, Bilbao, 48007 Spain; 2 GoiEner, Ordizia, 20240 Spain

**Keywords:** Energy modelling, Energy and behaviour

## Abstract

Smart meters are devices that provide detailed information about the energy consumed by specific electricity supply points, such as homes, offices, and businesses. Data from smart meters are useful for modeling energy systems, predicting electricity consumption, and understanding human behavior. We present the first smart meter dataset from Spanish electricity supply points, expanding the geographic diversity of available data on energy consumption at the household level and reducing biases in existing data, which typically come from a limited number of countries. The dataset consists of 25,559 raw hourly time series with an average length of nearly three years, spanning from November 2014 to June 2022. It also includes three subsets obtained by segmenting and cleaning the raw time series data, each focusing on the periods before, during, and after the COVID-19 lockdowns in Spain. This dataset is a valuable resource for studying electricity consumption patterns and behaviors that emerge in response to different natural experiments, such as nationwide and regional lockdowns, nighttime curfews, and changes in electricity pricing.

## Background & Summary

Smart meters are advanced devices that measure and record the consumption of individual electricity supply points, such as homes, offices, and businesses. Unlike traditional meters, which require manual reading, smart meters can communicate electricity consumption data directly to the energy supplier, providing real-time information on energy usage. In addition to informing pricing and billing, and providing consumers with detailed reports on their energy consumption, the data collected by smart meters has proven to be extremely valuable for several purposes^1,2^, such as:

- **Load profiling**, by enabling the creation of specific load profiles that can be used for a variety of applications, including sizing photovoltaic systems^3,4^, evaluating energy management systems^5,6^, and designing electricity tariffs^7,8^.
- **Energy efficiency**, by identifying energy waste and inefficiencies in a building or facility^6,9,10^ and informing energy-saving strategies^11^.
- **Renewable energy integration**, by monitoring the integration of renewable energy sources into the energy grid^12^ and optimizing their use^13,14^.
- **Demand response**, by managing energy demand in real-time and balancing the energy supply and demand^15–17^, reducing the need for expensive peak generation.
- **Power system planning**, by making predictions about future energy demand^18–20^, helping energy companies to plan for the future and make informed decisions about investments in generation and transmission.
- **Design of policies and programs** to encourage energy conservation and promote the use of renewable energy sources, by understanding human behavior related to energy consumption^11,21,22^.

There are a number of smart meter datasets that are publicly or easily available to researchers. Some of the largest, with over 5,000 supply points, include those from *EDRP*^23^, *SGSC*^24^, *SERL*^25^, *ISSDA*^26^, *SAVE*^27^, and *Low Carbon London*^28^. These datasets often provide information on other related factors, such as appliance use, climate, geographic location, and socio-demographics. In addition, other smaller datasets exist, such as those from *Elergone*^29^, *METER*^30^, *NESEMP*^31^, and *NEEA*^32^, to name a few. However, the majority of these datasets come from Western, English-speaking countries with similar cultural characteristics, which can bias the conclusions drawn from the data, especially in the field of human behavior analysis.

The dataset we present here is the result of the research conducted within the EU-funded *WHY* project (Grant Agreement ID: 891943), which aims to implement causal models to quantitatively analyze the everyday decisions people make about energy consumption and their responses to interventions. *GoiEner*, a retail cooperative specializing in renewable energy and a partner in the *WHY* project, has provided anonymized hourly electricity demand data for the 25,559 customers (or supply points) in its database for research purposes. These supply points are located primarily in individual homes, but also, to a lesser extent, in retail and department stores, offices, industrial plants, and public facilities. No supply point serves more than one individual household at a time. The data covers all provinces in peninsular Spain, with a notable concentration of customers in the northern regions of the Basque Country and Navarre.

This dataset is a valuable resource for researchers. First, it is the first time that a smart meter dataset from Spain has been made publicly available, helping to reduce geographic bias in research. Second, it is one of the largest collections of time series data available, having been collected from November 2014 to June 2022, with an average time series duration of approximately three years. Furthermore, the extended time frame of the dataset is particularly relevant for studying the electricity consumption patterns of households, businesses, and industries in response to various natural experiments that occurred during this period. These include nationwide lockdowns imposed during the first wave of the COVID-19 pandemic, regional lockdowns and nighttime curfews in subsequent waves, and the introduction of a new national electricity pricing system in June 2021.

Our dataset validation process successfully confirmed the presence of the three expected seasonal patterns typically observed in electricity consumption time series: daily, weekly, and annual^33,34^, which we specifically examined across selected sectors. This validation confirms the effectiveness of our data processing and the reliability of the dataset.

## Methods

The dataset described in this article consists of anonymized hourly electricity demand data from 25,559 electricity supply points. These supply points come from the customer database of GoiEner, an electricity cooperative founded in 2012 in the Basque Country, Spain, whose business model is based on renewable energy. This dataset was used to obtain part of the research results of the project “*Climbing the causality ladder to understand and project the energy demand of the residential sector*”, referred to as *WHY*. This project (Grant Agreement ID: 891943) was funded by the Horizon 2020 program of the European Union.

The main objective of the *WHY* project was to improve the modeling of energy demand in the major energy system models. This was achieved by implementing causal modeling and analyzing the energy consumption decisions made by individuals in their daily lives. The project aimed to develop innovative methods for short- and long-term load forecasting and to provide deeper insights into household energy consumption patterns. In order to develop the causal model, it was essential to have access to real electricity consumption data from the past and during the three years of the project (September 2020 to August 2023). This data was used to track changes over time and to profile different types of consumers. Over the course of the *WHY* project, behavioral changes were observed in response to unexpected interventions or events, including the emergency measures enacted due to the COVID-19 pandemic and changes in electricity tariff regulations.

### Data acquisition and collection

The original raw dataset provided by GoiEner consists of 71,048 files containing diverse information related to customer consumption, generation, contracted power, pricing, and other relevant data. These files were collected from *smart meters*, which are metering devices installed at customer supply points for processing electrical measurements. This meters are essential for billing users and managing contracts between distributors and retailers such as GoiEner, among other uses.

**Fig. 1:**
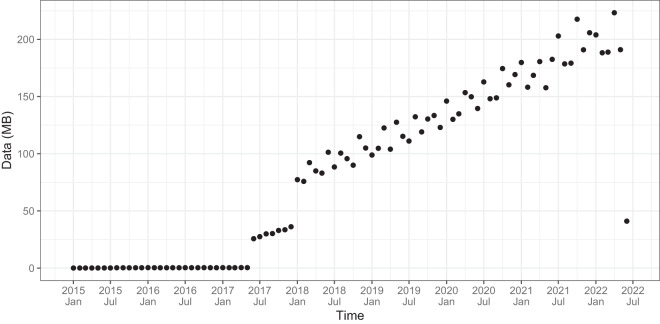
Data volume (in megabytes) of the GoiEner dataset over the collection period. The SIMEL files provided by GoiEner contain a total of 7.2 GB of information, covering the period from the end of 2014 to the beginning of June 2022. As shown in the figure, the use of smart meters in Spain did not become widespread until January 2018, when the main Spanish DSOs completed the deployment process. The increasing amount of information after this date corresponds to the growing number of customers that the company has experienced over the years. The outlier at the end of the series indicates that the last recorded month is not complete.

**Fig. 2:**
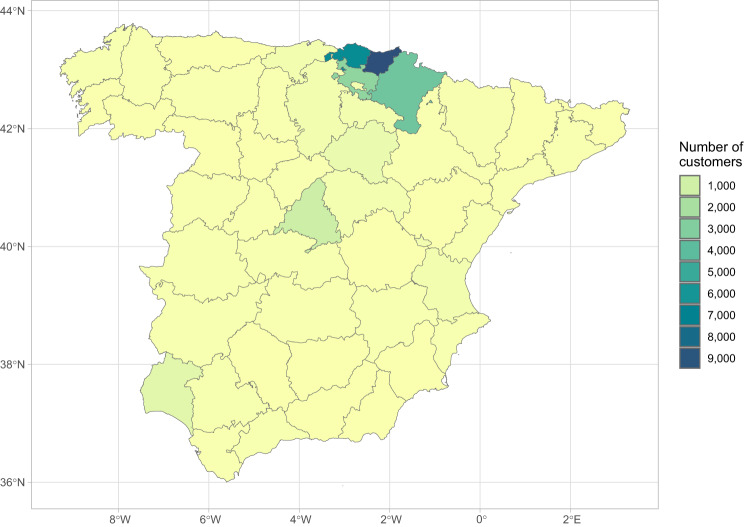
Number of GoiEner dataset records per province in peninsular Spain. They are mainly concentrated in the three provinces of the Basque Country (72.5%), Navarre (13.8%), and Madrid (4.4%).

The data collection period extends from the end of 2014 to June 2022, with a significant increase following the widespread deployment of smart meters since January 2018 (see Fig. 1). Furthermore, the dataset showcases significant diversity in terms of both geographical and economic aspects of the supply points recorded. In terms of geographical distribution, the dataset encompasses time series data from all the provinces of peninsular Spain, including both urban and rural municipalities. The majority of time series originate from the three provinces of the Basque Country (the place of origin of GoiEner), the neighboring region of Navarre, and the province of Madrid, as illustrated in the provincial map in Fig. 2.

**Fig. 3:**
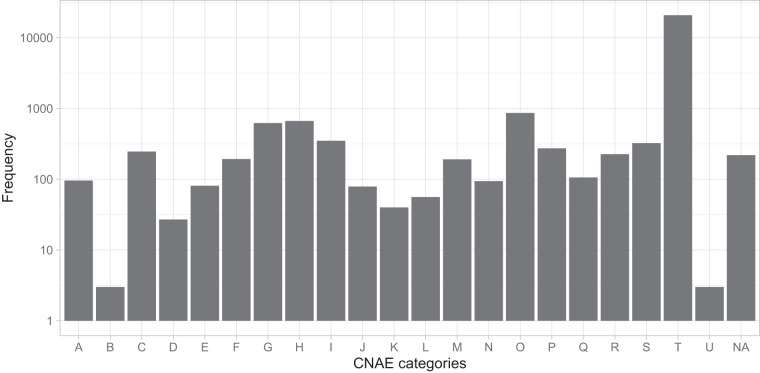
Number of time series belonging to each CNAE category (see Table 1) in logarithmic scale. *NA* indicates time series for which the CNAE category is not known.

**Fig. 4:**
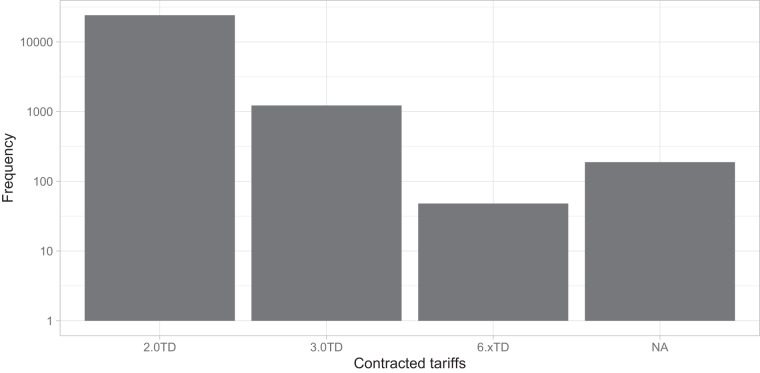
Number of time series belonging to each contracted tariff. Note the logarithmic scale on the vertical axis. *6.xTD* includes 6.1TD and 6.2TD. *NA* indicates time series for which the CNAE category is not known.

**Fig. 5:**
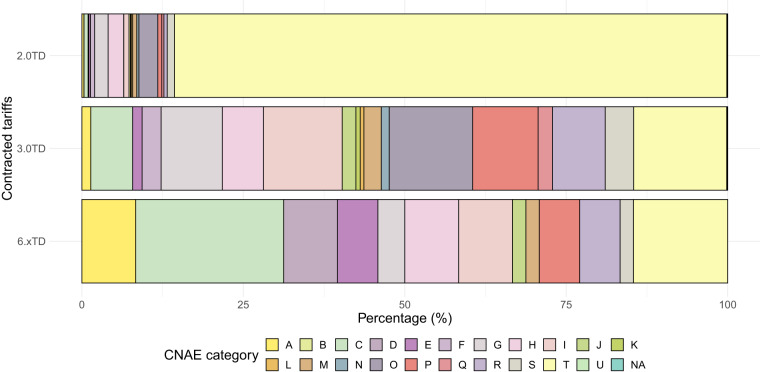
Percentage of categories per tariff.

Regarding the distribution of customers according to their economic activity, the metadata of the GoiEner dataset provides the *National Classification of Economic Activities* (CNAE) code for each customer (see the “Usage notes” section). Figure 3 presents the number of time series included within each of the 21 CNAE categories listed in Table 1. Notably, the category dedicated to household activities is the most prominent, followed by categories such as public administration activities, transportation and storage, wholesale and retail trade, and hospitality, each comprising more than 300 time series. All CNAE categories are represented in the dataset. In addition, the power contracted by customers is closely associated with the type of economic activity they engage in. Three types of contracted power are defined (see the “Usage Notes” section), with low-voltage contracts, as depicted in Fig. 4, being the most prevalent by a significant margin. Figure 5 further illustrates the number of time series by contracted power and economic activity, showing that lower levels of contracted power are typically associated with residential customers, while higher levels of contracted power are prevalent among industrial and energy-intensive enterprises.

**Table 1:** List of CNAE economic activity categories.

CNAE categ.	First digits	Economic activities
A	01-03	Agriculture, livestock, forestry, and fishing
B	05-09	Extractive industry
C	10–33	Manufacturing industry
D	35	Supply of electric power, gas, steam, and air conditioning
E	36–39	Water supply, sanitation, waste management, and decontamination
F	41–43	Construction
G	45–47	Wholesale and retail trade
H	49–53	Transportation and storage
I	55–56	Hospitality
J	58–63	Information and communications
K	64–66	Financial and insurance activities
L	68	Real estate activities
M	69–75	Professional, scientific, and technical activities
N	77–82	Administrative activities and auxiliary services
O	84	Public administration and defense
P	85	Education
Q	86–88	Health and social services activities
R	90–93	Artistic, recreational, and entertainment activities
S	94–96	Other services
T	97–98	Activities of households
U	99	Activities of extraterritorial organizations and bodies

**CNAE categ**.: CNAE category identifier. **First digits**: Interval in which the first two digits of the CNAE code for that category are found. **Economic activities**: The type of economic activities carried out for that category. For more details, please refer to the official table^54^. For example, in the CNAE 8411 code, the first two digits are 84, and therefore, that activity belongs to category “O” (*Public administration and defense; compulsory social security*).

Access to metering files is regulated by Spanish law, which gives both distribution system operators and retailers the right to access them. Therefore, the data is not publicly available unless an agreement is reached with the relevant parties. Within the *WHY* project consortium, an agreement has been reached to allow access to the data for research purposes and subsequent open publication.

In the contracts between customers and GoiEner, the sharing of *personal data* with third parties is strictly regulated and only allowed when required by law or with the explicit consent of the customer. However, it is important to note that data can be considered *non-personal* if it is completely anonymized and cannot be traced back to any individual. As a result, contractual restrictions do not apply to anonymized data, and there is no requirement for consent or the ability to revoke the publication of such data. This approach ensures compliance with Spanish (LOPDGDD) and European (GDPR) legislation on data protection and privacy.

To ensure a high level of data anonymization, GoiEner and the University of Deusto consulted their respective legal advisors and implemented several measures. First, personal data, including names, addresses, telephone numbers, and other identifiable information, were completely removed from the dataset. In addition, following the recommendations of legal advisors, supply point identifiers (CUPS) were replaced with unique 64-digit hexadecimal SHA-2 hash codes to prevent re-identification of individuals from the dataset. Furthermore, the dataset excludes names and postcodes of municipalities with a population of less than 50,000 to further protect privacy.

The customers of GoiEner were informed about all the activities carried out in the *WHY* project and were given the opportunity to opt out. The procedure was supervised by the Research Ethics Committee of the University of Deusto, which deemed it as ‘*FAVORABLE*’.

If other third parties are interested in using Spanish smart meter data, whether with GoiEner or any other retailer in Spain, they should contact the company directly for more information.

### From raw data to a fully processed dataset

The raw files provided by GoiEner adhere to the formats and specifications set by the *National Commission of Markets and Competition* (CNMC). As the regulator of the Spanish electricity market, the CNMC is responsible for monitoring the *Electricity Metering Information System* (SIMEL), which serves as the standard for formatting and structuring the data collected by smart meters. This guarantees that all files comply with the necessary standards and can be readily understood by all relevant parties.

Generating load profiles for all customers from the GoiEner files is a complex task that requires careful attention to the various file formats and strict adherence to the official protocols and standards established by the SIMEL. This process involves a detailed analysis of the technical peculiarities (see “SIMEL files”), as well as the development of a robust data processing scheme (see “SIMEL data processing scheme”). In addition, standard data cleaning procedures must be applied to the extracted time series data (see “Cleaning the raw data”).

### SIMEL files

The data files provided by GoiEner follow the SIMEL format, which includes over 140 file types for different types of data, such as load curves, inventory information, incidents, and billing^35^. Our primary interest is in extracting load curve data, which includes smart meter measurements of electricity consumption and self-generation for each customer over time.

The SIMEL type of a file can be determined by examining the first characters of its filename, which follows the format type_codes_date.v. In this format, type represents the file type; codes consists of up to two four-digit codes identifying the electricity distributor and/or retailer; date indicates the file creation date in YYYYMMDD format; and v is the version number of the file, starting from 0, which distinguishes files created on the same day. For example, a file named P5D_0021_1377_20220224.5 indicates that it is a file of type P5D. The code 0021 refers to the electricity distributor, while 1377 refers to the electricity retailer (in this case, GoiEner or one of its sister companies). The date of data collection is February 24, 2022, and the suffix indicates that this is the sixth file (since counting starts at 0) in a series of files with the same file name. The documentation does not clearly state how many versions of the same file can exist, or why new versions are created. It does mention, however, that the size of these copies is determined by the amount of information they contain, and that to ensure that all information for a given day is available, all versions of a file must be kept^35^.

**Fig. 6:**
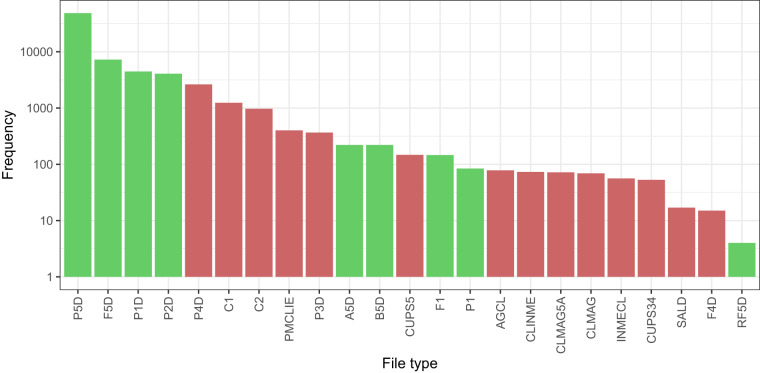
Number of GoiEner files by SIMEL file type, in logarithmic scale. By far the most common file type is P5D. The green bars indicate that the file type contains load curves, i.e. electricity consumption data. For further details on file types, see Table 2).

In Fig. 6, a log-scale graph shows the distribution of the SIMEL files by type. The most common file type is P5D with 68.1% of the files. The next most common file type is F5D with 10.2% of the dataset, followed by P1D with 6.3%. In total, the files provided by GoiEner contain 23 different types, but not all of them are defined by the SIMEL. Four file types (C1, C2, A5D, B5D) were created by the CNMC and were not included in the SIMEL at the time of analysis^36^. Similarly, three file types (P4D, P3D, F4D) were still being tested and had no publicly available information.

Table 2 provides pertinent details about the 23 file types that are among the files provided by GoiEner. To ensure accurate processing, we will only consider files categorized as *load curves* (i. e. P5D, F5D, P1D, P2D, A5D, B5D, F1, P1, and RF5D types), as these are the files that contain electricity consumption data. Although these file types share similar characteristics, there are subtle differences that are not critical for performing load curve extraction. For example, the P5D, F5D, and RF5D file types all contain hourly data from Type-5 customers (see Table 3). However, P5D files consist of data validated by the electricity distributor, while F5D files do not. Rather, they provide insight into how the data was collected, i. e. the acquisition method. In contrast, the data in RF5D files relate to changes in previous values after a claim. The methodology for processing the relevant SIMEL files is described in the following section.

**Table 2:** Technical details on the SIMEL file types.

File type	Number of files	Format descript.	Category	Samples per hour	Customer type	Self-cons. cust. type
P5D	48,422	SIMEL^35^	Load curves	1	5	
F5D	7,258	SIMEL	Load curves	1	5	
P1D	4,454	SIMEL	Load curves	1	1, 2, 3	4
P2D	4,078	SIMEL	Load curves	4	1, 2	
P4D	2,630	*Unpub*.				
C1	1,240	CNMC^36^	Contracting			
C2	967	CNMC	Contracting			
PMCLIE	401	SIMEL	Inventory			
P3D	366	*Unpub*.				
A5D	220	CNMC	Load curves	1		5
B5D	220	CNMC	Load curves	1		5
CUPS5	147	SIMEL	Inventory			
F1	146	SIMEL	Load curves	1	1, 2, 3	
P1	84	SIMEL	Load curves	1	1, 2, 3	4
AGCL	78	SIMEL	Inventory			
CLINME	73	SIMEL	Aggregate meas.			
CLMAG5A	72	SIMEL	Aggregate meas.			
CLMAG	69	SIMEL	Aggregate meas.			
INMECL	56	SIMEL	Aggregate meas.			
CUPS34	53	SIMEL	Inventory			
SALD	17	SIMEL	Billing			
F4D	15	*Unpub*.				
RF5D	4	SIMEL	Load curves	1	5	

**File type**: The type of SIMEL file. **Number of files**: The quantity of files of each type among the files provided by GoiEner. **Format descript**.: Reference to the format description of each file type (*Unpub*. means unpublished information). **Category**: Information contained in each file type (load curves, contracting information, inventory, aggregated measurements, billing). We focus on analyzing load curve files to obtain electricity consumption data. **Samples per hour**: Sampling frequency of electricity consumption data. **Customer type**: Type of customer for which the data are recorded, as defined by the Spanish electricity system (refer to Table 3). **Self-cons. cust. type**: The customer type recorded if they have the ability to consume their own generated energy.

**Table 3:** Classification of metering points and boundaries of the Spanish electrical system^57^.

Customer type	Contracted power *p*	Commonly addressed to
5	*p* ≤ 15 kW	household consumers, small businesses
4	15 < *p*≤50 kW	office buildings, larger businesses, small factories
3	any other case	—
2	*p* > 450 kW	larger factories, shopping malls, hospitals, government buildings
1	*p* ≥ 10 MW	large industrial consumers: power plants, refineries, etc.

In the case of customer boundaries, this classification is determined by the contracted power *p*. Examples are given of the type of customer addressed on the basis of their power contracted.

### SIMEL data processing scheme

SIMEL files store data in plain ASCII text lines, with each line consisting of a series of semicolon-separated fields that vary greatly depending on the file type. Fields are denoted by letters, starting with A and continuing alphabetically. However, not all file types and fields are relevant for extracting electricity consumption or self-generation data. Table 4 summarizes the relevant fields for load curve file types, which are the only ones that contain electricity usage data.

**Table 4:** Summary of the most relevant fields per SIMEL file type categorized as *load curves*^35,36^.

File type	MP	DT	FL	IN	OUT	DCM
A5D	A	B	C	D [Wh]	E (*empty*)	J
B5D	A	B	C	D [Wh]	E [Wh]	J
F1	A	C	D	E	F	M
F5D	A	B	C	D [Wh]	E [Wh]	J
P1	A	C	D	E	G	U
P1D	A	C	D	E	G	U
P2D	A	C	D	E	G	U
P5D	A	B	C	D [Wh]	E [Wh]	(*none*)
RF5D	A	B	C	D [Wh]	E [Wh]	J

**MP**: measurement point code (CUPS). **DT**: date and time of the measurement in the format “yyyy/mm/dd hh:mm”. **FL**: a binary summer/winter flag indicating whether the daylight saving time is on for that time. **IN**: “measurement of the incoming active value”, i.e. the energy consumed by the CUPS in an hour. The default units are kWh, unless otherwise stated. **OUT**: “measurement of the outgoing active value”, i.e. the energy generated by the CUPS in an hour. The default units are kWh, unless otherwise stated. **DCM**: “data collection method”. It can take any value between 1 and 6, with 1–3 being firm measurements and 4–6 being provisional measurements. As a rule of thumb, the lower the value of this field the more reliable the measurements.

One such field is the *Unified Supply Point Code* (CUPS), a 20 to 22-character alphanumeric code that uniquely identifies Spanish electricity supply points. To comply with Spanish and European personal data protection legislation, all CUPSs in the SIMEL files have been anonymized using unique 64-digit hexadecimal SHA-2 hash codes. Other fields in the SIMEL files include the timestamp, which indicates the date and time when the data was recorded, and the values of electricity consumed from the grid and supplied to the grid. Typically, a single SIMEL file contains electricity usage data for multiple CUPS.

Our goal is to extract all relevant information (anonymized CUPS, timestamps, and electricity usage data for both consumption from the grid and supply to the grid) from each SIMEL file and reorganize it into a CSV file per customer. To achieve this, we have defined three processing steps: (1) processing the SIMEL files to obtain a single file per CUPS, i. e. the CUPS files; (2) eliminating duplicate entries in the CUPS files; and (3) cleaning the raw data to generate the electricity consumption CSV files.

### From SIMEL files to CUPS files

**Fig. 7:**
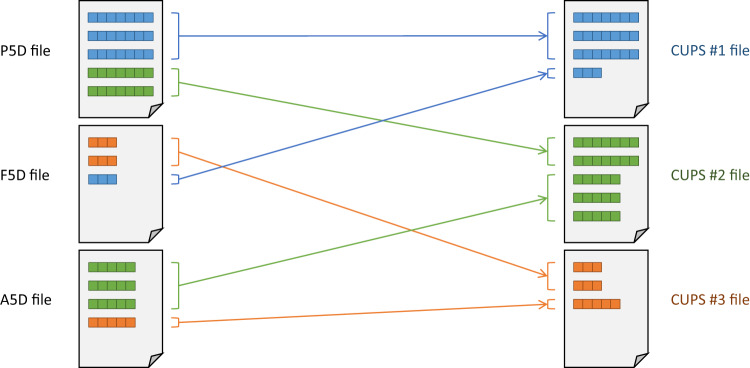
SIMEL files (left) are processed to generate files with unique CUPSs (right). Lines with different CUPS are indicated with different colours and lines with different formats are indicated with different line lengths.

The first step is to create individual files for each anonymized CUPS by collecting its corresponding entries from all SIMEL files, as shown in Fig. 7. To do this, we copy all entries related to a particular anonymized CUPS from any SIMEL file into its corresponding CUPS file. To ensure traceability, we prepend the name of the original SIMEL file as a new field to each copied entry in the CUPS files. The result is a collection of CUPS files, each containing all the entries that belong to the same anonymized CUPS. Since the CUPS files consist of entries from different SIMEL files, and therefore different SIMEL file types, the entries usually have different formats.

To avoid redundancy, the P2D files have been excluded from this process, as they contain the same information as the P1D files, but in a more detailed format (quarter-hourly instead of hourly data, see Table 2). This approach ensures the homogeneity of the dataset by using consistent 1-hour samples across all files.

### Elimination of duplicate entries in the CUPS files

The purpose of this step is to locate and correct any instances of duplicate entries, that is, those with the same date and time. There are many reasons why this occurs. For example, when a smart meter is unable to connect to its operator, it may transmit estimated usage data that may be updated later. This results in multiple entries with the same timestamp but conflicting information. Other duplicates are generated due to the nature of SIMEL files. For example, A5D and B5D files contain complementary information about self-consumed and feed-in electricity, respectively.

Regarding self-generated electricity, certain GoiEner customers can generate their own electricty, typically through photovoltaic panels or batteries. These customers are required to maintain a connection to the general grid to manage any excess or deficit in their self-generated electricity. Within the CUPS files, two fields are relevant at this stage of data processing: IN, denoting *incoming active values*, and OUT, representing *outgoing active values* (see Table 4). The IN field records data about electricity consumption, regardless of whether it originates from the grid or self-generated sources. Conversely, the OUT field records data on feed-in electricity, which refers to the electricity supplied back to the grid by customers producing surplus energy. For the specific requirements of the *WHY* project, this dataset focuses exclusively on electricity consumption by customers, regardless of its source. As a result, only the values within the IN field are considered, along with the fields that provide the timestamp.

As a general approach, duplicate timestamps do not require disambiguation if they contain the same IN values, regardless of the file type. In almost 95% of cases with duplicate timestamps, all duplicate entries provide the same IN value. However, if duplicate timestamps contain different values, further analysis is required. Thus, if one of the duplicate entries comes from a P5D file, its IN values are assumed to be correct because P5D files contain *validated raw* data^35^. This situation accounts for about 5% of the cases with duplicate timestamps.

There are also other less common cases (<0.4%) that need to be taken into consideration as well:

1. All load curve file types (except P5D, which is already *validated raw* data) must specify how the recorded values were obtained by an integer value in the “*data collection method*” field (see Table 4). The range of integers varies for each file type, with values closer to 1 indicating more reliable firm measurements and more distant values indicating estimates. Therefore, if repeated entries come from the same file type but have different data collection methods, the correct value is the one whose “*data collection method*” value is closest to 1. If multiple values meet this criterion, the average of all of them is calculated.
  In the case of duplicate entries for A5D and B5D file types, the IN value represents self-consumed electricity and is always taken from the A5D entry. This is because, in practice, the B5D IN value is set to 0. However, if there are additional F5D or P1D file types combined with A5D or B5D files, which is the typical scenario, the electricity values from the F5D and P1D files are preferred since they already consolidate all the information provided by the A5D and B5D files. If there are multiple entries from F5D or P1D files, the entry with the “*data collection method*” value closest to 1 is selected. If there are still multiple entries after this selection, their average is calculated.
2. If there are repeated entries from RF5D file types, their values are considered correct over the values from other file types. This is because the RF5D files are created according to claims. If there are multiple duplicates from RF5D files, their average is calculated.
3. Finally, if the duplicate entries do not meet the above criteria, the average of the IN values of all duplicate entries is calculated.

After processing all entries in the CUPS files to ensure unique timestamps and unique electricity consumption values, the entries are sorted from oldest to newest. This process generates a raw CSV file comprising a time series of electricity consumption data for each CUPS file. Depending on the specific application, these raw files can serve as a valuable starting point; however, the subsequent section will detail the necessary further processing steps to address potential issues.

### Cleaning the raw data

At this point, all load profiles have been extracted from the SIMEL files provided by GoiEner and saved as raw CSV files of electricity consumption. However, these files do not yet meet the requirements for a comprehensive validation analysis, as they may contain gaps with missing values, data inconsistencies, or outliers that need to be corrected and harmonized. This process is commonly referred to as data cleaning. In this section, we outline the transformation of the **raw CSV files of electricity consumption** into **fully processed files**. To do this, we perform three key operations, namely: (1) data imputation; (2) adjustment to local time; and (3) segmentation of the time series into three periods related to the COVID-19 lockdowns.

#### Data imputation

Two different data imputation strategies are used, depending on the length of the missing value sequences. For missing value sequences of eight consecutive hours or less, linear interpolation is used to impute the missing values. For missing value sequences longer than eight consecutive hours, the Last Observation Carried Forward (LOCF) method with a 7-day season is used. This means that missing samples are replaced with values from the previous seven days, thus keeping the same time and day of the week. If the missing value sequences are longer than seven days, the same sequence is repeated as many times as necessary. If missing samples are at the beginning of the time series where there are no previous seven days of data, they are replaced with values from the nearest seven-day period without missing values.

#### Adjustment to local time

Time series data from countries that observe Daylight Saving Time (DST) are often challenged by the clock changes. DST, which aims to make better use of daylight and conserve energy, involves moving clocks forward one hour in the spring and back in the fall. This adjustment can cause inconsistencies in data sets. For example, there is often a missing data point when time goes from 2:00 to 3:00 in the spring, and a duplicate timestamp when it goes back from 3:00 to 2:00 in the fall.

To address these issues and simplify future analysis, our dataset does not adjust for Daylight Saving Time (DST). Instead, we fill in missing data and average repeated data. This method helps keep the data consistent and accurate throughout the year, making it easier to analyze and interpret. Note that this approach is not the same as using Coordinated Universal Time (UTC); it simply maintains local time without DST modifications. By treating DST as non-existent, our approach effectively resolves the data inconsistencies caused by clock changes.

#### Time series segmentation

In early 2020, several countries enforced lockdowns to stop the spread of COVID-19, which greatly changed the way people used electricity at home. This dataset is segmented into three parts, each representing different stages of the pandemic in Spain, to make it easier to compare and analyze: (1) **before lockdowns**: this includes the regular data up to two weeks before the lockdowns started on March 1, 2020; (2) **during lockdowns**: this includes data from the start of the lockdowns on March 1, 2020, to May 30, 2021, including the peak of the pandemic; and (3) **after lockdowns**: this part includes data from May 30, 2021, after the lockdown restrictions have eased and life has returned to normal.

## Data Records

The original anonymized SIMEL files provided by GoiEner are available on Zenodo, in the “*GoiEner smart meters raw data*” repository^37^. Through the processing steps described in the previous sections, it is possible to extract the electricity usage CSV files prior to data cleaning (see “SIMEL data processing scheme”) and the three subdatasets corresponding to periods before, during, and after lockdowns (see “Cleaning the raw data”). These four large datasets are also available on Zenodo in the “*GoiEner smart meters data*” repository^38^. The latter also includes a metadata file that provides relevant information for each entry in any of the three subdatasets. All files are publicly available and licensed under the *Creative Commons Attribution 4.0 International License*. A detailed description of the contents of the repositories is provided below.

- The “*GoiEner smart meters raw data*” repository^37^ consists of a single file named GoiEner.zip with a size of 8.0 GB. This file contains 71,048 SIMEL files, each compressed in gzip format as provided by the source. The distribution of file collection dates can be found in Fig. 1, while the distribution of SIMEL file types is shown in Fig. 6.
- The “*GoiEner smart meters data*” repository^38^ contains five files, the largest of which are compressed using the Zstandard compression algorithm:

1. raw.tzst: The initial file size is 2.0 GB and consists of a folder containing 25,559 CSV files, totaling 15.1 GB when uncompressed. Each file in the folder is named using a 64-digit hexadecimal number that serves as an anonymized representation of a GoiEner customer. These files store time series data related to electricity consumption or generation, and have been processed directly from the original SIMEL files. Note that these data have undergone the processing steps described in the “SIMEL data processing scheme” section, but have not yet undergone the data cleaning process described in the “Cleaning the raw data” section.
2. Each time series may cover a different collection interval, depending on the duration of the customer’s relationship with the company, and therefore may have a different length. The files may contain gaps due to missing samples. The data are structured in two columns with no named headers. The first column indicates the timestamp of each record, while the second column indicates the customer’s electricity consumption in kWh.
3. imp-pre.tzst: This file is 791.6 MB in size and contains a 6.28 GB folder with 12,149 CSV files when uncompressed. The filenames are 64-digit hexadecimal numbers, each representing an anonymized GoiEner customer. These files contain processed time series data of electricity consumption of customers of GoiEner, and have been processed from the files contained in the raw.tzst file. Each time series may have a different length and cover a different collection interval, but all of them have a minimum duration of one year and all data have been collected before March 1, 2020, i. e. before the Spanish COVID-19 lockdowns. The data are structured in three named columns: *timestamp*, *kWh* and *imputed*, the latter being a binary column indicating whether the rows were obtained by imputation.
4. imp-in.tzst: This file is 555.9 MB in size and contains a 4.36 GB folder with 15,562 CSV files when uncompressed. The contents are similar to those described for the imp-pre.tzst file, but are specifically focused on the Spanish COVID-19 lockdown period, i. e. between March 1, 2020 and May 30, 2021.
5. imp-post.tzst: This file is 508.2 MB in size and contains a 4.01 GB folder with 17,519 CSV files when uncompressed. The contents are similar to those described for the imp-pre.tzst file, but are specifically focused on the period after the Spanish COVID-19 lockdowns, i. e. after May 30, 2021.
6. metadata.csv: This CSV file is 5.6 MB in size and provides metadata (see Table 5) for the 25,559 anonymized GoiEner customers included in the preceding data files.

**Table 5:** Explanation of the fields in the metadata.csv file.

Field	Contents
user	64-digit hexadecimal number representing an anonymized CUPS.
start_date	First timestamp of the time series.
end_date	Last timestamp of the time series.
length_days	Number of days between the first and last timestamp.
length_years	Number of years between the first and last timestamp.
potential_samples	Number of samples in the time series, assuming no missing values.
actual_samples	Actual number of samples in the time series.
missing_samples_abs	Number of missing samples.
missing_samples_pct	Percentage of missing samples out of total number of samples.
contract_start_date	Start date of the contract with GoiEner.
contract_end_date	End date of the contract with GoiEner.
contracted_tariff	Type of the contracted tariff (*2.X*, *3.X* or *6.X*; see “Usage Notes”).
self_consumption_type	Type of self-consumption (see “Usage Notes”).
p1, p2, p3, p4, p5, p6	Contracted power (in kW) for each of the six time slots (see “Usage Notes”).
province	Spanish province where the Unified Supply Point is located.
municipality	Spanish municipality (>50,000 inhabitants) where the Unified Supply Point is located.
zip_code	Spanish postal code (>50,000 inhabitants) where the Unified Supply Point is located.
cnae	CNAE code for the economic activity of the customer (see “Usage Notes”).

## Technical Validation

All stages of our methodology incorporate robust mechanisms to ensure the quality of the resulting dataset. The following sections describe these procedures in more detail. In addition, we provide insight into the reliability of our dataset by comparing the generated time series with behavioral and electricity consumption patterns described in other studies.

### Before data cleaning

When the SIMEL files are processed to obtain the CUPS files (see “From SIMEL files to CUPS files”), an additional column is added to all CUPS files to identify the source SIMEL file for each entry, serving as a traceability measure. This traceability proved to be crucial in the early stages of processing to verify the correct creation of CUPS files from the corresponding SIMEL file types. Indeed, a rigorous examination of the technical details of all file types (see Table 2) was conducted to select only those categorized as “load curves” containing reliable electricity consumption data. This selection process excluded unpublished file types such as P3D, P4D, and F4D, even though they may contain useful fields. The decision to exclude them was based on the lack of official documentation, which may result in a loss of guaranteed reliability.

The workflow outlined in the “Elimination of duplicate entries in the CUPS files” section presents a logical sequence of decisions aimed at selecting the appropriate electricity consumption value among duplicate timestamps in the CUPS files. This process inherently includes a layer of data reliability verification. At each step, if none of the specified conditions is met, the conflicting sample is determined by averaging all duplicate values rather than deleting them. For simplicity, it is not possible to trace back all candidate values after the estimate has been consolidated. However, it is important to note that this situation only occurs in a small fraction of cases where there are conflicting duplicate timestamps. This approach is based on the assumption that having an approximate estimate of the value is preferable to relying on a future imputation of the deleted value.

Another important aspect is P2D files. These files have a sampling frequency that is four times higher than the rest of the file types. When these values are aggregated to hourly samples, which is the desired sampling frequency, the resulting values are the same as those found in P1D file types. Therefore, values from P2D file types are excluded from further processing to avoid redundancy and conflicts in cases where P2D files are missing.

**Fig. 8:**
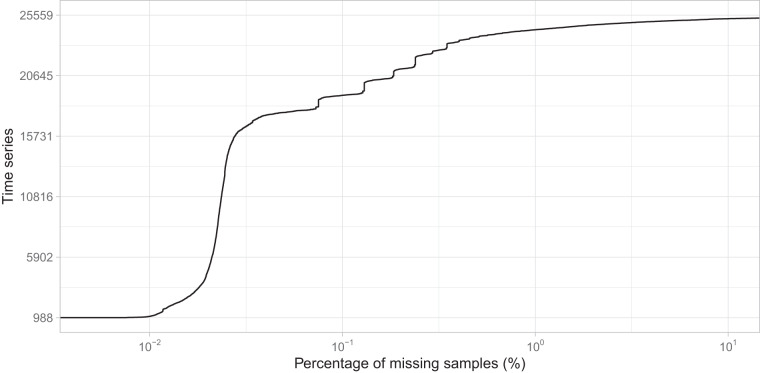
Cumulative distribution of time series by percentage of missing samples.

**Fig. 9:**
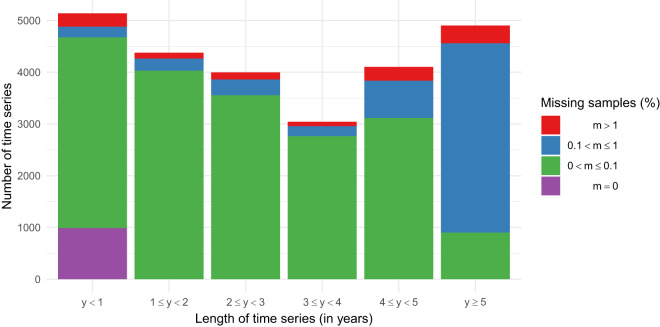
Missing samples by time series length.

The time series contained in the raw.tzst file have been stored prior to the data cleaning process described in the “Cleaning the raw data” section. Consequently, no imputation processing has been applied to these time series, which may contain some gaps due to missing samples. Figure 8 illustrates that the incidence of missing samples in the dataset is relatively low. Among the time series, about 95.3% (24,368 series) have less than 1% missing samples, with about 74.5% (19,047 series) having less than 0.1% missing samples. In addition, there are 988 time series with no missing samples at all. On the other end, 109 time series have more than 50% missing samples, with 15 of them having more than 90% missing samples. Building on this analysis, Fig. 9 examines the distribution of missing samples based on the length of the time series. It shows that time series with a length of five years or more have a higher proportion of missing data, while all time series with no missing data have a length of less than one year.

To facilitate the reusability of the time series within the raw.tzst file, we included information about the number of missing samples in the metadata.csv file (see Table 5). This additional metadata complements the information provided by GoiEner, such as contract details and geographic data. The number of missing samples is reported both as absolute values in the field “*missing_samples_abs*” and as a percentage in the field “*missing_samples_pct*‘”. To calculate these values, we first determined the expected number of samples in each time series using the initial and final time stamps (“*start_date*” and “*end_date*” fields, respectively). We then counted the actual number of samples in each time series (“*actual_samples*” field). The absolute number of missing samples is derived by subtracting the actual samples from the potential samples, while the percentage of missing samples is calculated by dividing the absolute number of missing samples by the potential number of samples and multiplying by 100. This approach provides flexibility to users, allowing them to choose how to impute data or select time series based on their preferences.

### During data cleaning

The time series contained in the imp-pre.tzst, imp-in.tzst, and imp-post.tzst files are derived from the data in the raw.tzst file, but have undergone the data cleaning process described in the “Cleaning the raw data” section. This process includes imputation of missing values and adjustment to local time as described in that section. In addition, the data have been split into three subsets corresponding to the periods before, during and after the COVID-19 restrictions. This approach ensures the reusability of the data for comparative analyses between these three different periods. It also allows for the possibility of conducting analyses excluding the period of pandemic-related restrictions, which had a significant impact on household electricity consumption patterns.

Building upon this, additional quality assessments were performed on the time series within these files. First, we performed imputation for all missing samples, as described in the “Data imputation” section. To increase user flexibility, we included a Boolean integer column in the time series CSV files that indicates whether imputation was applied to the entries. This allows users to select their own preferred imputation methods, if desired.

Second, although rare, we encountered some time series where all values were consistently zero. These cases were excluded at this stage of data processing because they can introduce errors in certain analyses. For example, calculations involving division operations with values derived from such time series, are particularly problematic. This includes operations such as min-max scaling, standardization, coefficient of variation, and signal-to-noise ratio, among others.

Finally, following the segmentation process described in the “Time series segmentation” section, all time series within each subset with a duration of less than one year were removed. This decision is consistent with models and analyses that typically rely on the three primary seasonalities commonly found in electricity consumption time series: daily, weekly, and annual^39–42^. As a result, about 52.5% of GoiEner customers in the pre-pandemic subset, about 39.1% of customers in the pandemic subset, and about 31.5% of customers in the post-pandemic subset were removed due to their shorter time series. It is important to note that the length, and start and end dates of the time series recorded for a particular customer will determine whether it appears in none, one, two, or all three of the segmented subsets.

### Consumption pattern analysis

**Fig. 10:**
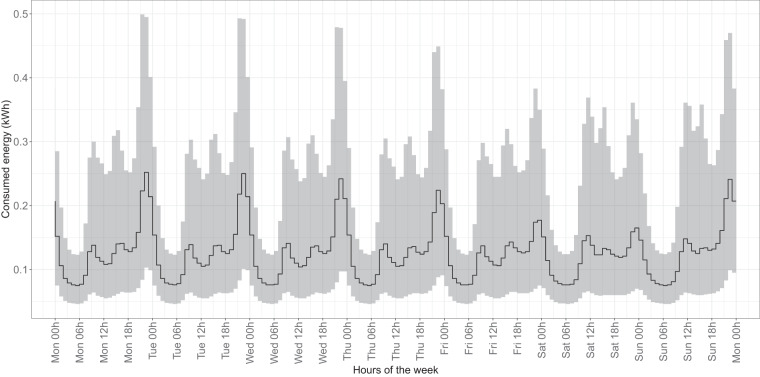
Median and confidence interval (1st and 3rd quartiles) of electricity consumption in kWh for all hours of the week in 2018 and 2019 for all time series belonging to CNAE category T (households).

**Fig. 11:**
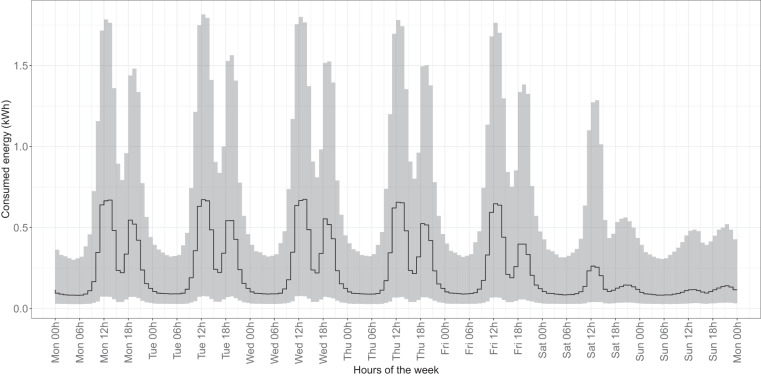
Median and confidence interval (1st and 3rd quartiles) of electricity consumption in kWh for all hours of the week in 2018 and 2019 for all time series belonging to CNAE categories C (industry) and G (commerce).

**Fig. 12:**
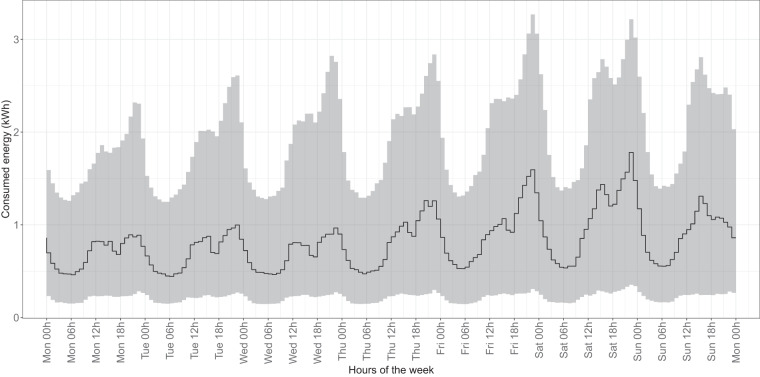
Median and confidence interval (1st and 3rd quartiles) of electricity consumption in kWh for all hours of the week in 2018 and 2019 for all time series belonging to CNAE category I (hospitality).

**Fig. 13:**
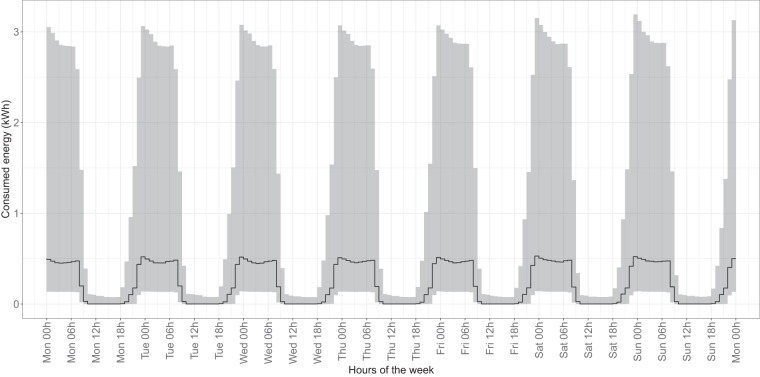
Median and confidence interval (1st and 3rd quartiles) of electricity consumption in kWh for all hours of the week in 2018 and 2019 for all time series belonging to CNAE category O (public administration, street lighting).

To assess the verifiability and reliability of our dataset, we conducted baseline comparisons with previously published studies of electricity consumption patterns in Spain. The time series of electricity consumption that we generated closely matches established consumption patterns observed in various sectors, including residential (Fig. 10), industrial and commercial (Fig. 11), hospitality (Fig. 12), and public administration (Fig. 13).

Focusing on specific sectors, for example, in the residential sector, we observed typical peak consumption times corresponding to daily routines such as morning preparations, afternoon cooking, and evening leisure activities^43,44^. Similarly, the commercial and industrial sector showed distinct patterns, with pronounced daytime consumption during working hours and a noticeable drop during traditional shift work, in line with Southern European working patterns^45,46^.

Additionally, weekend variations in the residential and hospitality sectors also reflect common social practices, with shifts in activity times and increased nighttime energy use, particularly in the hospitality sector during leisure and entertainment activities^47,48^. In addition, public administration consumption, primarily driven by municipal lighting, showed a regular pattern consistent with the availability of natural light.

In terms of annual patterns, the years leading up to 2020 provide a stable baseline for potential analyses, showing typical daily and weekly behavior with seasonal variations and reduced electricity consumption during holidays, as observed in our dataset^39^. However, the patterns in 2020 show significant deviations due to the effects of the COVID-19 pandemic (see “Usage Notes”). Lockdowns and curfews led to a noticeable increase in electricity consumption, especially in the afternoon and evening, accompanied by a shift in nighttime consumption^33,49,50^. These changes, as seen in our dataset, were particularly pronounced during the strict lockdown periods and continued with subsequent curfews until 2021, reflecting the adaptation of the population to new routines and restrictions^51,52^.

These observations confirm the alignment of our dataset with well-established daily, weekly, and annual consumption patterns, thereby reinforcing its reliability as a reflection of real-world behavior.

## Usage Notes

Overall, the usability of the dataset is straightforward. It comprises time series data containing comma-separated values of timestamps and energy consumption (or generation) values. Consequently, the data can be easily analyzed using various data-centric programming languages, including R, Python, or Matlab.

It is important to note that the raw.tzst file contains data for all customers in the GoiEner database, regardless of the duration of their recorded data. However, the datasets in the imp-pre.tzst, imp-in.tzst, and imp-post.tzst files have specific criteria for including customers. A customer will only appear in these datasets if they have at least one year of recorded data for the corresponding interval. As a result, a customer may appear in all, some or none of the datasets.

As for the data that appears in the metadata file, metadata.csv, there are four fields (see Table 5) that require further clarification:

- **contracted_tariff**: In Spain, the access tariffs for domestic electricity use have undergone changes. Prior to June 2021, there were several types of contracts: 2.0 A, 2.0DHA, 2.0DHS, 2.1 A, and 2.1DHA, which corresponded to different tariff structures. However, since June 2021, these tariffs have been consolidated into a single tariff called 2.0TD. The 2.0TD tariff applies to power ratings up to 15 kW (low voltage). For small and medium-sized companies with contracted power above 15 kW (low voltage), the old tariff was 3.0 A. However, this tariff has been replaced by the 3.0TD tariff since June 2021. Additionally, the previous tariff 3.1 A, which was designed for medium and high voltage installations with a maximum power of 450 kW, has been merged with tariff 6.1TD. Previously, tariff 6.1 A was used for access to contracted power above 15 kW (high voltage). In June 2021, this tariff was replaced by the new access tariff, 6.XTD^53^. In the metadata file, this field shows the last recorded tariff.
- **self_consumption_type**: The code provided classifies customers based on their self-consumption type. There are five categories represented by the following identifiers: “0” and “00” indicate no self-consumption, while “41”, “42”, and “43” indicate self-consumption with surplus energy and corresponding economic compensation. In the metadata file, most of the entries lack this value, indicating that they do not have self-consumption.
- **p1,** **p2,** **p3,** **p4,** **p5,** **p6**: The new electricity tariffs, introduced in June 2021, define a series of periods for transmission and distribution charges. These tariffs introduce six consumption periods: P1, P2, P3, P4, P5 and P6. Among these periods, P1 is the most expensive, while P6 is the least expensive. The metadata file contains information about the contracted power for each of these periods (1, 2, 3, 4, 5, and 6). The contracted power values increase progressively from period 1 to period 6, i. e. it is not possible to have a contracted power for period 6 that is lower than the contracted power for period 1^53^.
- **cnae**: The *National Classification of Economic Activities* (CNAE) codes are four-digit codes used in Spain to classify and group economic activities carried out by companies and institutions. The official table of CNAE codes provides a description of these activities and establishes their corresponding European NACE codes^54^. Within the dataset, the majority of users (81.4%) are associated with codes starting with “98,” which indicates households. The next most prevalent code is “84” (3.4%), which represents public administration, followed by code “52” (2.5%), which represents storage and other transport activities.

It is also important to note that Spain experienced a significant impact from the COVID-19 pandemic in 2020. This impact resulted in the implementation of strict home lockdowns, curfews, and movement restrictions, which had a profound impact on the electricity consumption patterns that can be derived from our dataset. For additional context, we have included a timeline of key pandemic-related policies below for reference^55,56^.

- On **14 March 2020**, at the start of the *first wave* of the pandemic, a general lockdown was implemented throughout Spain, with exceptions for essential activities such as obtaining basic necessities and fulfilling work obligations. Educational, cultural, and leisure facilities were closed.
- On **30 March 2020**, the lockdown was tightened to include workers in non-essential sectors.
- On **10 April 2020**, workers in non-essential sectors were allowed to return to work.
- On **26 April 2020**, children under the age of 14 were allowed to be outside near their homes for one hour per day.
- On **2 May 2020**, outdoor exercise was permitted in time slots based on age and location within one kilometer of home.
- On **11 May 2020**, a phased and regional plan to lift restrictions and transition to a *new normal* was initiated.
- On **21 June 2020**, the lockdown officially ended, and the *new normal* began.
- On **25 October 2020**, during the *second wave* of the pandemic, a curfew was imposed beginning at 10 pm-12 am and ending at 5–7 am, depending on the region. There were also restrictions on freedom of movement, with lockdowns at or below the municipal level depending on the epidemiological situation.
- On **9 May 2021**, the curfew and movement restrictions were lifted.

## Data Availability

The code files used to process the dataset provided by GoiEner are publicly available on GitHub (https://github.com/DeustoTech/GoiEner-dataset) and are licensed under the GPL-3.0. The repository includes a comprehensive README.md file with detailed information and instructions on how to run the code. The code is written in R version 4.2.2 and is compatible with both Windows and Linux operating systems.
